# Proteome-Wide Analysis of Functional Phosphosites in the FGFR Family of Proteins: Insights from Large-Scale Phosphoproteomic Analysis

**DOI:** 10.3390/proteomes14010008

**Published:** 2026-02-13

**Authors:** Akhina Palollathil, Althaf Mahin, Athira Perunelly Gopalakrishnan, Tejaswini R Poojari, Alimath Sambreena, Prathik Basthikoppa Shivamurthy, Rajesh Raju

**Affiliations:** Centre for Integrative Omics Data Science (CIODS), Yenepoya (Deemed to be University), Mangalore 575018, Karnataka, India; akhinap.ciods@yenepoya.edu.in (A.P.); althafmahin99@gmail.com (A.M.); athirajrf@yenepoya.edu.in (A.P.G.); tejaswinirpoojari.ciods@yenepoya.edu.in (T.R.P.); sambreenaibrahim75@gmail.com (A.S.); prathikbsgowda@gmail.com (P.B.S.)

**Keywords:** cancer, mass spectrometry, phosphoproteomics, phosphorylation, post-translational modification, receptor tyrosine kinases, skeletal dysplasia

## Abstract

Background: Fibroblast growth factor receptors (FGFRs) play a crucial role in tissue homeostasis and organ development by regulating cellular processes, including proliferation, differentiation, and survival. Dysregulation of FGFRs contributes to developmental disorders and carcinogenesis. As membrane-bound receptors, they represent promising targets for therapeutic intervention and drug development. Methods: This study employed a systematic in silico analysis of publicly available phosphoproteomics datasets to provide a comprehensive overview of the phosphorylation regulatory network of the FGFR family. Results: We identified predominant phosphosites in FGFR1-4 that exhibited differential abundance across diverse experimental conditions, specifically, Y653 in FGFR1; S453, Y586, Y656, and Y657 in FGFR2; S444 and S445 in FGFR3; and S573 in FGFR4. Our analysis identified 32 and 89 significantly co-modulated phosphosites on other proteins with FGFR3 and FGFR4, respectively. Beyond the upstream kinases from the FGFR family, we also identified MAPK1 as a potential upstream kinase of FGFR4. Furthermore, disease enrichment analysis revealed that proteins co-modulated with FGFR3 were primarily involved in skeletal developmental disorders, such as brachydactyly, short toe, and syndactyly of fingers, whereas those associated with FGFR4 were linked to various cancers. Conclusions: Our findings highlight key disease-associated phosphosites within the FGFRs and offer a foundation for advancing phosphosite-focused therapeutic research.

## 1. Introduction

Receptor tyrosine kinases (RTKs) are an important subfamily of transmembrane proteins that bind extracellular ligands specific to their family and initiate downstream signaling cascades [1]. Fibroblast growth factor receptors (FGFRs) represent a subcategory of RTKs that possess a molecular weight ranging from 17 to 34 kDa in vertebrates [2,3]. The FGFR family comprises five members (FGFR1-5), which share common structural and functional characteristics [4]. They are located on human chromosomes 8 (FGFR1), 10 (FGFR2), 4 (FGFR3), and 5 (FGFR5), respectively [5]. Structurally, FGFRs are single-pass transmembrane proteins composed of three main regions: an extracellular domain, a transmembrane domain (TMD), and an intracellular tyrosine kinase domain [6]. The extracellular ligand-binding domain consists of three immunoglobulin (Ig)-like folds and an acidic box. The TMD anchors them in the cell membrane and facilitates lateral dimerization. The juxtamembrane region of FGFRs contributes to receptor dimerization in the cytosol and promotes intermolecular transphosphorylation, a crucial step for activating downstream signaling [6]. Unlike the other four members, FGFR5 (known as FGFRL1) lacks a tyrosine kinase domain [3]. FGFRs are activated by a family of ligands known as fibroblast growth factors (FGFs). In humans, 18 canonical FGFs specifically bind to FGFRs in a ligand-receptor-dependent manner [7,8].

Dysregulation of FGFR signaling plays a vital role in carcinogenesis and developmental disorders [9]. FGFRs have been reported as an oncogene that promotes the development and progression of multiple cancers, including lung squamous cell carcinoma [10], breast cancer [11], and head and neck squamous cell carcinoma [12]. Aberrant FGFR2 activation has been associated with poor prognosis characterized by increased lymph node metastasis and tumor invasion in gastric cancer [13,14], endometrial cancer [15], and colorectal adenocarcinoma [16]. In addition to mRNA and protein level overexpression of FGFRs and their ligands in cancers and skeletal disorders such as chondrodysplasia syndromes and craniosynostosis syndromes [17,18,19,20], aberrant phosphorylation also emerges as a key regulatory mechanism driving FGFR-associated pathology. Studies have reported that sequential and precisely ordered autophosphorylation of FGFR1 at Y653, Y583, Y463, Y766, Y585, and Y654 is crucial for stimulating its kinase activity and the subsequent phosphorylation of downstream substrates [21,22]. However, mutations such as FGFR1 N546K, located within the kinase domain, can disrupt this ordered autophosphorylation sequence, leading to enhanced kinase activity. This dysregulated activation has been shown to drive glioblastoma progression [22]. A previous study has demonstrated that chronic phosphorylation of FGFR1 at Y766 and the associated PLCγ signaling pathways is critical for the gradual, age-related acquisition of proliferative capacity in nonmalignant prostate tumor epithelial cells [23]. Targeting this specific pathway has been suggested as a potential strategy to prevent the progression of latent tumors into aggressive, malignant prostate cancer [23]. Upon activation, FGFR3 and FGFR4 subsequently initiate MAPK and PI3K-AKT signaling cascades [24], thereby promoting cellular proliferation, survival, angiogenesis and motility in hepatocellular carcinoma [25,26]. FGFR2 L770V mutation reduces phosphorylation at Y769, a critical site for PLCγ binding, thereby impairing activation of the PLCγ signaling pathway in melanoma [27]. Mass spectrometry-based analysis of a panel of 24 triple-negative breast cancer cell lines revealed overexpression and enhanced phosphorylation of FGFR2 at Y656/Y657 and FGFR3 at Y577/Y599/Y647 [28]. However, further experimental validation is required to elucidate the functional significance of these phosphorylation events.

Furthermore, next-generation sequencing analysis of 4853 solid tumors delineated the spectrum of FGFR alterations, predominantly comprising gene amplifications (66%), followed by mutations (26%) and rearrangements (8%). FGFR1 exhibited the highest frequency of alterations (3.5% of patients), with progressively lower frequencies observed for FGFR3 (2.0%), FGFR2 (1.5%), and FGFR4 (0.5%) [29]. The most frequently detected mutation in FGFR1 is N546K, which is associated with neuroblastoma [30]. N546K mutation triggers autophosphorylation and catalytic activity of FGFR1, leading to progression of Ewing sarcoma and brain tumors [31,32]. Several FGFR2 mutations have been identified across multiple cancer types, including breast cancer (R203C, N550K, S588C, K660M), cervical cancer (A97T, S252L, P256S, K406E), lung cancer (Q621K, R626T, D138N, N211I, D247Y, D283N, W290C, G302W, S320C, E471Q), oral cancer (V393A, G272V, P253R), gastric cancer (S267P, Q212K, G463E), and colorectal cancer (R203H, R210Q, D334N, Q361R, L552I). FGFR2 mutations were also detected in skeletal disorders such as apert syndrome (M186T, P252S/W/F/L, P253L/R, S267P), Crouzon syndrome (A315T/S, A344G/P, C278F, F276V, G338R, K526E), and Pfeiffer syndrome (A172F, A314D, C278F, C342F/R/S, K641R, N549D/K, S267P) [33]. The R203C and K660N mutation in FGFR2 increases the kinase activity of FGFR2 in breast cancer [34]. FGFR3 has been identified as a key protein associated with various skeletal dysplasias, including achondroplasia, hypochondroplasia, thanatophoric dysplasia (types 1 and 2), and severe achondroplasia with developmental delay and acanthosis nigricans (SADDAN) [35]. Achondroplasia is the most common form of dwarfism, characterized by disproportionate short stature. Over 97% of individuals with achondroplasia carry a p.Gly380Arg mutation in the transmembrane domain of the FGFR3 gene [36]. Hypochondroplasia is a mild form of achondroplasia that often arises due to the p.Asn540Lys mutation in FGFR3. Thanatophoric dysplasia is predominantly associated with the FGFR3 p.Arg248Cys mutation, whereas the FGFR3 p.Lys650Met mutation is implicated in SADDAN [37,38]. To combat FGFR-driven aberrations in various forms of cancer, ongoing efforts focus on a range of treatments, including small-molecule inhibitors that target the kinase domain, ligand traps that block FGF, and monoclonal antibodies that engage with the extracellular domain of FGFR [18,39]. Clinical trials have evaluated the efficacy of first-generation FGFR inhibitors, including lucitanib, dovitinib, ponatinib, anlotinib, derazantinib, and nintedanib, for conditions where FGFR mutations are implicated. As these are non-selective tyrosine kinase inhibitors, they inhibit other kinases in addition to FGFRs. Second-generation FGFR inhibitors have improved potency, safety, selectivity, and unique modality. Erdafitinib, pemigatinib, and infigratinib are the three FDA-approved inhibitors in this class, while numerous other drugs are undergoing preclinical and clinical testing [39].

Considering the pivotal role of FGFRs in developmental abnormalities and oncogenic processes, understanding their phosphoregulatory landscape is crucial for deciphering their involvement in disease mechanisms and for developing targeted therapeutic interventions. Proteoforms-centric phosphosite analyses provide a more precise representation of signaling events than gene-centric approaches, as phosphosites in proteoforms act as the functional units that directly influence cellular signaling pathways by regulating protein stability, localization, and protein–protein interactions [40]. The concept of proteoforms highlights the complexity of protein biology, as individual proteins can exist in multiple molecular variants arising from alternative splicing, sequence polymorphisms, and the combinatorial effects of post-translational modifications (PTMs) [41]. Mass spectrometry-based bottom-up and top-down strategies represent the two primary analytical approaches for identifying proteoforms. The bottom-up proteomics provides high sequence coverage and site-specific localization of PTMs within peptides; however, the peptide-centric nature imposes significant limitations, as peptides resulting from protein digestion are often difficult to assign to a specific proteoform. Moreover, enzymatic cleavage also disrupts information on sequence variants and abolishes the connectivity between PTMs and their precise positions within proteoforms. In contrast, top-down proteomics introduces intact proteins directly into the mass spectrometer, preserving sequence integrity and the full PTM landscape, thereby enabling direct proteoform identification. Nevertheless, this approach is constrained by limited depth and throughput [41]. Recent studies have introduced various novel strategies to improve the proteoform identification using bottom-up proteomics by integrating additional techniques and advanced data analysis pipelines [40,42]. Although bottom-up phosphoproteomics is limited in resolving complete proteoform architectures, phosphosite-level analyses nevertheless provide valuable insights into the PTMs at the isoform level. Hence, in this study, we utilized phosphoproteomics datasets reporting FGFR1-5 phosphorylation to identify functionally significant phosphosites across the FGFR family of proteins. An in silico pipeline was applied to systematically identify the predominant phosphosites in FGFR1-5, the positively and negatively co-modulated phosphosites on other proteins, potential upstream kinases and phosphatases of FGFRs, and the disease associations of the co-modulated proteins.

## 2. Materials and Methods

### 2.1. Phosphoproteomics Data Mining for FGFR1-5 Phosphosites

Publicly available human cellular phosphoproteomics data was mined from PubMed using the following search terms: “phosphoproteomics” OR “phosphoproteome” NOT “Plant” NOT “Review”. The data were curated, integrated, and processed using an in silico pipeline described in our previous work [43,44]. Briefly, class I phosphosites on FGFR1-5 (localization probability ≥ 75%, A score > 13) were selected and categorized based on the phosphopeptide enrichment method used (STY, ST, or Y). Subsequently, significantly altered phosphoproteins (*p* < 0.05) with a fold change ≥1.3 or ≤0.76 were considered as increased or decreased abundance. To ensure uniformity, protein identifiers were standardized by mapping to HGNC gene symbols and UniProt accession numbers. Each dataset was coded using standardized experimental and biological descriptors to facilitate downstream analysis.

### 2.2. Identification of Predominant Phosphosites in FGFR1-5

The class I FGFR phosphosites identified from the profiling and differential abundance datasets were ranked based on their detection frequency across different experimental conditions. Class I phosphosites that were consistently detected at high abundance across datasets and exhibited significant differential modulation were defined as predominant sites. Predominant phosphosites in FGFRs were selected for downstream analysis, whereas low-frequency phosphosites were excluded [43,44].

### 2.3. Detection of Phosphosites on Other Proteins Co-Modulated with FGFR1-5

Identification of co-modulated proteins is a powerful approach for investigating the functions of proteins with limited functional annotation. As phosphosite-specific functions of FGFRs remain poorly characterized, we adopted a co-modulation analysis strategy to gain insights into their functional roles. As the present study utilized a large number of phosphoproteomics datasets, which cover a wide array of experimental conditions, biological contexts, and analytical platforms, it was not feasible to reanalyze the raw data. Therefore, we relied on the data as reported in the original studies. The processing and downstream analyses were performed following the systematic methodology established in our previous work, as follows. To identify phosphosites on other proteins (PsOPs) that are positive and negative co-modulation with each predominant FGFR1-5 phosphosite, the differential abundance datasets were utilized. Based on the abundance status of FGFR phosphosites and PsOPs, the differential datasets were categorized into four groups. Datasets where both the FGFR site and PsOP show increased abundance were categorized as UU, whereas FGFR site with increased abundance and PsOP with decreased abundance was labeled as UD. Conversely, data with both FGFR site and PsOP exhibiting decreased abundance were marked as DD, with FGFR is showing decreased abundance, while PsOP showing increased abundance was categorized as DU. Furthermore, datasets in which both predominant sites and PsOPs showed similar abundance trends were classified as positively co-modulated (UUDD, by combining UU and DD datasets), whereas negative co-modulation (UDDU, combining UD and DU pairs) was defined for phosphosites exhibiting opposite abundance trends [43,44].

To minimize bias arising from datasets containing an excessive number of phosphosites from one or two studies, or from multiple time points of a single stimulus, we applied stringent data filtering criteria. A one-sided Fisher’s Exact Test (FET) was performed to evaluate the likelihood and confidence of co-modulation patterns, and those with FET *p*-values < 0.05 were considered significant. The significantly co-modulated phosphosite pairs were further filtered based on the ratio of ∑(nUU + nDD)/∑(nUD + nDU) for positive patterns, ∑(nUD + nDU)/∑(nUU + nDD) for negative patterns. A ratio exceeding 10% of the total differential frequency was considered high-confidence and selected for further processing. The high-confidence co-modulated phosphosites were further filtered based on PMID and experimental codes (conditions sharing the same stimulus were grouped under one experimental code). Positive and negative experimental code confidence scores were computed as UUDD EXP Code—UDDU EXP Code and UDDU EXP Code—UUDD EXP Code, respectively. PMID confidence was determined by the number of unique studies reporting the co-modulation. Phosphosites with both experimental code and PMID confidence scores ≥ 3 was considered highly significant and retained for further analysis [43,44].

### 2.4. Compilation of Interactors and Upstream Kinases of FGFR3/4

The publicly available protein–protein interaction repositories, including IntAct (European Bioinformatics Institute (EMBL-EBI), Cambridge, United Kingdom) [45], BioGRID (The Biological General Repository for Interaction Datasets, Toronto, Ontario, Canada) [46], HPRD (Human Protein Reference Database, Institute of Bioinformatics, Bangalore, India) [47], and BIND (Biomolecular Interaction Network Database, Blueprint Initiative, Toronto, Ontario, Canada) [48], were utilized to fetch the data on interactors of FGFR3 and FGFR4 (retrieved on 22 May 2023). Experimentally validated upstream kinases of FGFR3 and FGFR4 were retrieved from PhosphoSitePlus (Cell Signaling Technology, Danvers, MA, USA), (retrieved on 22 May 2023) [49], Phospho.ELM 9.0 (European Bioinformatics Institute (EMBL-EBI), Hinxton, Cambridge, United Kingdom), (retrieved on 24 May 2023) [50], and RegPhos 2.0 (Taiwan, China), (retrieved on 24 May 2023) [51]. Predicted kinase associations were gathered from NetworKIN (retrieved on 4 January 2023) (Samuel Lunenfeld Research Institute, Mount Sinai Hospital, Toronto, Canada) [52], AKID (retrieved on 24 May 2023) [53], and iKiP-DB (Berlin) [54]. In addition, kinase-substrate specificity data with ≥ In confidence, derived from synthetic peptide library profiling by Johnson et al. (2023), were included [55].

### 2.5. Functional Characterization of Co-Modulated Proteins

The protein and mRNA level expression of FGFR1-5 in human tissues and organs were assessed by utilizing the protein abundance and RNA-seq data from the Human Protein Atlas (https://www.proteinatlas.org/), (Stockholm, Sweden), (accessed on 18 December 2025). The phosphosite-level abundance patterns of FGFR3 and FGFR4 were analyzed using the CProSite database (https://cprosite.ccr.cancer.gov/), (National Cancer Institute, Bethesda/Rockville, MD, USA), (accessed on 19 December 2025). Phosphosite-specific functions of co-modulated phosphoproteins were explored by using the PhosphoSitePlus database [49]. The DisGeNET database (Barcelona, Spain) was used to enrich the diseases associated with proteins positively and negatively co-modulated with FGFR3 and FGFR4 [56].

### 2.6. Data Visualization

The TrackViewer package in RStudio, (version 4.2.2) was employed to generate lollipop plots [57]. Matplotlib (version 3.10.0) in Python 3.8.20 was used to depict co-differential frequencies of PsOPs. Circular and linear dendrograms were created with RAWGraphs, (version 2.0) (https://www.rawgraphs.io/). The bubble plots were generated using the ggplot2 package in RStudio, (version 4.2.2) (https://ggplot2.tidyverse.org/). The comparative analysis was performed by creating a Venn diagram using the online tool, InteractiVenn [58]. The FASTA sequences of FGFR 1-4 isoforms were downloaded from UniProt, and sequence conservation across the protein isoforms was analyzed using the Clustal Omega Multiple Sequence Alignment (MSA) tool, (EMBL–EBI, Hinxton, Cambridge, UK) (https://www.ebi.ac.uk/jdispatcher/msa/clustalo (accessed on 19 December 2025).

## 3. Results

### 3.1. Organ-Specific Gene and Protein Expression of FGFR1-5

FGFRs are a family of receptor tyrosine kinases and play essential roles in organ development and tissue homeostasis. Among the five members of the FGFR family, FGFR1-4 share substantial sequence similarity and are capable of initiating downstream signaling upon ligand binding, whereas FGFR5 lacks an intracellular kinase domain and is therefore unable to transduce downstream signals [59]. Initially, we examined the mRNA and protein-level expression patterns of FGFR1–5 across human organs using transcriptomic and proteomic data from the Human Protein Atlas database. At the transcript level, FGFR1 exhibited low tissue specificity and was expressed across nearly all human organs. At the protein level, elevated FGFR1 abundance was observed in the esophagus, gallbladder, fallopian tube, and placenta, while most organs displayed moderate expression. FGFR2 showed tissue-specific mRNA expression in the brain, particularly in the spinal cord; however, this pattern was not reflected at the protein level. Instead, higher FGFR2 protein abundance was detected in the nasopharynx, bronchus, oesophagus, and testis. Increased FGFR3 mRNA expression was observed in the skin, whereas protein-level expression was restricted to a limited number of organs, with high abundance detected in the hippocampus, skin, and tonsil. FGFR4 displayed elevated mRNA expression in the liver and lungs, with corresponding protein enrichment in the liver and additional organs of the digestive system. In contrast, FGFR5 did not exhibit tissue-specific expression, as both mRNA and protein were detected across most human organs. These expression patterns highlight the tissue-specific roles of FGFRs, particularly FGFR2, FGFR3, and FGFR4, and provide a foundation for understanding their contributions to normal physiology and disease (Supplementary Figure S1).

### 3.2. Integration of Phosphoproteomics Datasets to Identify Predominant Sites in FGFR1-5

**Figure 1:**
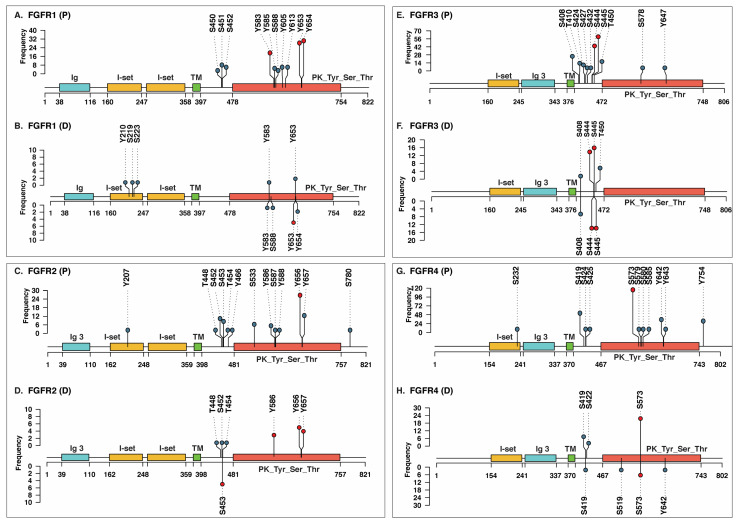
The frequency of phosphorylation at serine (S), threonine (T), and tyrosine (Y) residues is markedly higher in the canonical isoforms of fibroblast growth factor receptor 1-4 (FGFR1–4). Class-I phosphosites identified from phosphoproteomics profiling datasets for (**A**) FGFR1, (**C**) FGFR2, (**E**) FGFR3, and (**G**) FGFR4. Class-I phosphosites identified from differential abundance datasets for (**B**) FGFR1, (**D**) FGFR2, (**F**) FGFR3, and (**H**) FGFR4. The nodes with green color represent a specific phosphosite, while red represents the predominant phosphosite. (P): Profiling, (D): Differential abundance.

We next examined the phosphorylation landscape of FGFRs, as site-specific phosphorylation regulates receptor activation, subcellular localization, and protein interactions, and its dysregulation has been linked to cancer and developmental disorders. To identify the frequently detected phosphosites in FGFRs, a PubMed search was carried out. The PubMed search retrieved around 3800 phosphoproteomic datasets, among which datasets containing information on phosphorylation of FGFR1-5 were selected for downstream analysis. A total of 80, 40, 121, and 167 profiling datasets (Supplementary Table S1A–D) and 10, 10, 40, and 43 differential abundance datasets corresponding to FGFR1, FGFR2, FGFR3, and FGFR4 were identified, respectively (Supplementary Table S2A–D). Due to the lack of profiling and differential datasets for FGFR5, it was excluded from downstream analysis. Through systematic analysis of the profiling datasets, we identified 10, 13, 10, and 11 previously characterized phosphosites in the canonical isoforms of FGFR1, FGFR2, FGFR3, and FGFR4, respectively. In the differential abundance datasets, 7, 7, 4, and 5 phosphosites were detected in the respective canonical isoforms. Specifically, Y653, Y654, Y583, S588, Y210, S219, and S223 were detected in FGFR1; S453, Y656, Y657, Y586, T448, S452, and T454 in FGFR2; S444, S445, T450, and S408 in FGFR3; and S573, S419, S422, S519, and Y642 in FGFR4 (Figure 1A–H).

**Figure 2:**
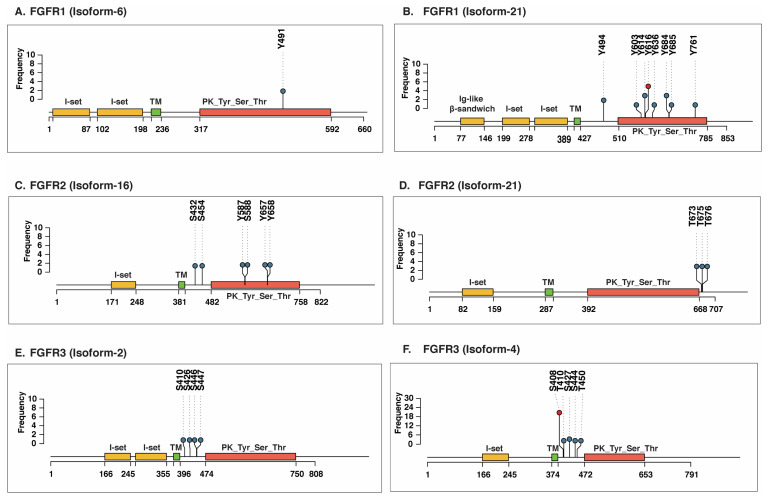
Reduced detection of phosphorylation at serine (S), threonine (T), and tyrosine (Y) residues in the non-canonical isoforms of FGFR1-3, based on phosphoproteomics profiling studies. Class-I phosphosites in (**A**) FGFR1 isoform-6, (**B**) FGFR1 isoform-21, (**C**) FGFR2 isoform-16, (**D**) FGFR2 isoform-21, (**E**) FGFR3 isoform-2, and (**F**) FGFR3 isoform-4. The nodes with green color represent a specific phosphosite, while red represents the predominant phosphosite.

As proteins often exist in multiple isoforms and undergo distinct phosphorylation events, this results in the generation of functionally diverse proteoforms. To understand this diversity, we examined phosphosite information at the isoform level for FGFRs. Identifying PTMs at the isoform level is crucial, as different proteoforms of the same protein can exhibit distinct biochemical activities, signaling outputs, and disease associations [40]. From the profiling data, we observed phosphosite information for FGFR1 isoform-6, FGFR1 isoform-21, FGFR2 isoform-16, FGFR2 isoform-21, FGFR3 isoform-2, and FGFR3 isoform-4 (Figure 2). The phosphosites identified in FGFR1 isoform-6 include Y491, while isoform-21 includes Y494, Y603, Y614, and Y616 (Figure 2A,B). Likewise, six and three phosphosites were observed in FGFR2 isoform-16 (S432, Y587, S588, Y657, S454, and Y658) and isoform-21 (T673, T675, and T676), respectively (Figure 2C,D). The FGFR3 isoform-2 exhibited phosphorylations at S426, S410, S446, and S447, whereas the isoform-4 exhibited phosphorylation at S408, S427, S444, T410, and T450 (Figure 2E,F). For FGFR4, phosphosite information was available only for the canonical isoform, with no data detected for other isoforms in our integrated dataset.

To examine the phosphosite conservation across all the isoforms of FGFR1-3, a multiple sequence alignment was performed using the Clustal Omega tool (Supplementary Table S2E–H). The result showed that some of the phosphosites were conserved across the isoforms. For instance, the Y654 phosphosite in canonical FGFR1 was conserved across FGFR1 isoform-6 (at Y491) and FGFR1 isoform-21 (Y684). We also found phosphosites that are unique to a particular isoform, such as S450/451/452/588 and Y613 in canonical FGFR1, which were not observed in other isoforms. Likewise, unique and conserved phosphosites were observed in isoforms of FGFR2 and FGFR3 (Table 1). As these isoform-specific PTMs hold functional relevance, further studies are required to confirm their isoform-specificity and biological significance. Notably, when we compared the frequency of phosphosite detection across the FGFR isoforms, it was observed that the number of phosphosites and their frequency of detection were high in the canonical isoforms of FGFR1-4 as compared to other isoforms.

Furthermore, we ranked all phosphosites identified in canonical FGFR1-4 based on their detection frequency across multiple experimental conditions. Phosphosites that are consistently high abundance across datasets, and exhibiting significant differential modulation were considered predominant sites. From the profiling datasets, multiple predominant phosphorylation sites in FGFR1 (Y653, Y583, and Y654), FGFR2 (Y656), FGFR3 (S444 and S445), and FGFR4 (S573) were identified. Predominant phosphorylation sites identified from the differential abundance datasets include Y653 on FGFR1; S453, Y586, Y656, and Y657 on FGFR2; S444 and S445 on FGFR3; and S573 on FGFR4. In line with earlier reports, the predominant phosphosites in FGFR1, FGFR2 and FGFR4 were localized within the kinase domain [60]. In contrast, FGFR2 exhibited predominant sites both within and outside the kinase domain, whereas in FGFR3, the sites were primarily detected outside the kinase domain. Figure 1 depicts the complete set of phosphosites identified in FGFR1-4, the predominant sites among them, and their detection frequency across profiling and differential datasets. For subsequent downstream analyses, we focused on the predominant phosphorylation sites of FGFR1-4 with differential abundance, as they are more likely to have functional relevance under specific experimental conditions.

### 3.3. Phosphoproteins Co-Modulated with Predominant Phosphosites in FGFR1-4

**Figure 3:**
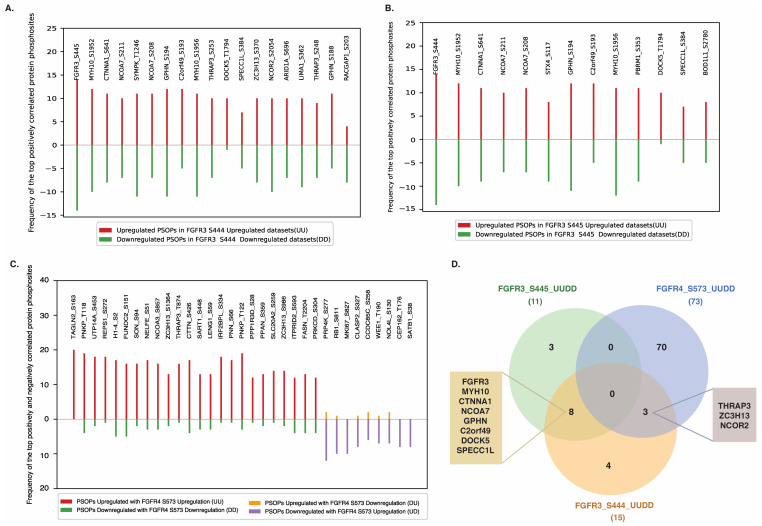
Phosphosites on other proteins (PsOPs) co-modulated with predominant phosphosites of FGFR3 and FGFR4 reveal extensive positive co-regulation. PsOPs positively co-modulated with (**A**) FGFR3 S444 and (**B**) FGFR3 S445. (**C**) PsOPs positively and negatively co-modulated with FGFR4 S573. (**D**) Proteins commonly co-modulated with FGFR3 and FGFR4 predominant sites. Eight proteins were commonly identified among those positively co-modulated with FGFR3_S445 and FGFR3_S444. Three proteins were shared between those positively co-modulated with FGFR4_S573 and FGFR3_S444.

It is well documented that co-modulated proteins often participate in related biological functions and signaling pathways. Such coordinated regulation also reflects signaling crosstalk. To explore the functional relevance of predominant sites in FGFR1-4, we examined their co-modulation pattern with PsOPs. The significantly positively and negatively co-modulated PsOPs were fetched from the phosphoproteomics differential abundance datasets using a significance cutoff of FET *p*-value < 0.05, with recurrent detection in at least three independent studies and under three or more distinct experimental conditions. Several PsOPs were identified to be co-modulated with predominant sites in FGFR1 and FGFR2; however, they did not meet the stringent criteria and were therefore excluded from further analysis. Only PsOPs co-modulated with the predominant sites on FGFR3 (such as S444 and S445) and FGFR4 (such as S573) passed the selection criteria. A total of 19 and 13 PsOPs, corresponding to 15 and 11 proteins, were positively co-modulated with FGFR3 predominant sites S444 and S445, respectively (Supplementary Table S3A,B). The top positively co-modulated PsOPs with FGFR3_S444 and S445 include GPHN_S194 and MYH10_S1956, among others (Figure 3A,B). FGFR3 predominant sites did not show any significant negative co-modulation with PsOPs. The predominant site S573 in FGFR4 exhibited positive co-modulation with 80 PsOPs (mapping to 73 proteins) and negative co-modulation with 9 PsOPs (mapping to 9 proteins) (Supplementary Table S4A,B). The top positively co-modulated PsOPs with FGFR4_S573 include PNKP_T118, H1-4_S2, and PNKP_T122, whereas the negatively co-modulated PsOPs include PRP4K_S277, RB1_S811, and MKI67_S827 (Figure 3C).

### 3.4. Overlapping PsOPs Co-Modulated with Predominant Sites in FGFR3 and FGFR4

A comparative analysis was conducted to identify PsOPs commonly co-modulated with the predominant sites of FGFR3 (S444, S445) and FGFR4 (S573). The analysis revealed that eight shared proteins carrying nine phosphosites were commonly co-modulated with both S444 and S445 of FGFR3. These included GPHN_S194, MYH10_S1952, MYH10_S1956, CTNNA1_S641, NCOA7_S208, NCOA7_S211, C2orf49_S193, SPECC1L_S384, and DOCK5_T1794. This suggests that the predominant sites of FGFR3 largely share the same set of co-modulated PsOPs. Additionally, while FGFR3 and FGFR4 exhibited overlap in co-modulated proteins (THRAP3, ZC3H13, and NCOR2), the specific phosphosites differed. For instance, the phosphorylation of THRAP3 at S253 and S248 was co-modulated with FGFR3, whereas T874 was co-modulated with FGFR4. Phosphorylation at S370 of ZC3H13 was co-modulated with FGFR3, while S1364 and S986 were co-modulated with FGFR4. In NCOR2, phosphorylation at S2054 was associated with FGFR3, whereas S2223 was associated with FGFR4 (Figure 3D).

### 3.5. Functional Categorization of Phosphoproteins Co-Modulated with FGFR3 and FGFR4

**Figure 4:**
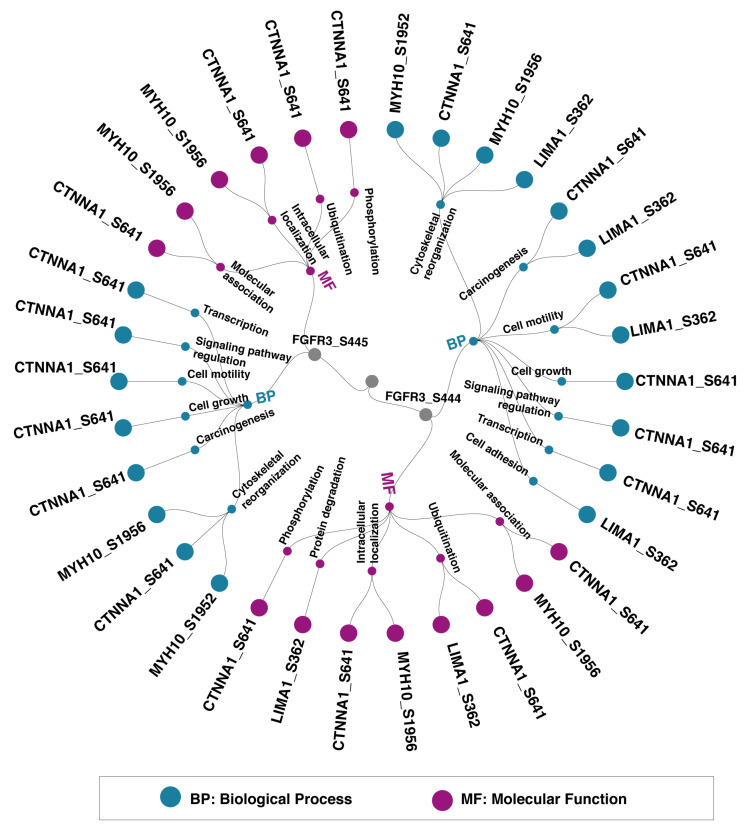
Phosphosite-level functional analysis of proteins co-modulated with FGFR3 reveals enrichment in functions linked to carcinogenesis. Molecular functions and biological processes of PsOPs positively co-modulated with the predominant sites on FGFR3 (S444, S445). The site-specific functions were obtained from the PhosphoSitePlus database.

**Figure 5:**
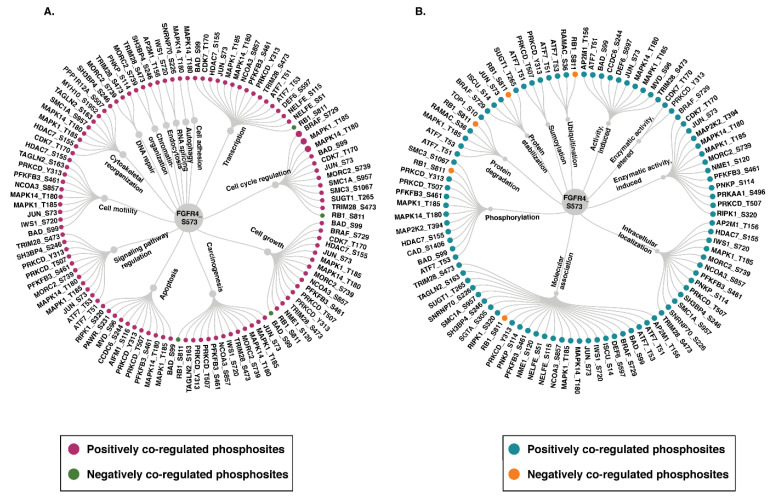
Phosphosite-specific functional annotation of proteins co-modulated with FGFR4 shows involvement in carcinogenesis related processes. (**A**) Biological processes and (**B**) molecular functions of PsOPs positively and negatively co-modulated with the predominant sites on FGFR4 (S573). The site-specific functions were obtained from the PhosphoSitePlus database.

As specific phosphosites can modulate distinct protein functions, the PhosphoSitePlus database was employed to identify the phosphosite-specific biological processes and molecular functions associated with PsOPs. The PsOPs positively co-modulated with FGFR3 S444 and S445 were found to be involved in biological functions such as cytoskeletal organization (CTNNA1_S641, MYH10_S1952, and MYH10_S1956), cell motility (CTNNA1_S641), and carcinogenesis (CTNNA1_S641 and LIMA1_S362). The molecular functions modulated by them comprised phosphorylation (CTNNA1_S641), molecular association (MYH10_S1956), and intracellular localization (CTNNA1_S641 and MYH10_S1956) (Figure 4). Furthermore, PsOPs positively co-modulated with FGFR4 S573, such as TAGLN2_S163, which influence the biological functions, such as carcinogenesis, cell motility, and cytoskeletal reorganization. MAPK1_T185 is involved in apoptosis, cell growth, and cell differentiation. JUN_S73 is associated with cell motility, signaling pathways, cell cycle, and transcription. RB1_S811, the negatively co-modulated PsOPs, was involved in carcinogenesis, cell growth, and cell cycle regulation. The molecular function of the positively co-modulated PsOPs of FGFR4 S573 comprised protein stabilization, molecular association, protein degradation, and enzymatic activity. Negatively co-modulated PsOPs include phosphorylation, protein stabilization, and ubiquitination (Figure 5).

### 3.6. Co-Modulation of FGFR3 and FGFR4 with Binary and Complex Interactors

To investigate the co-modulation of phosphosites on both binary and complex interactors of FGFR3 and FGFR4, interactome data were retrieved from multiple databases, including BioGRID, IntAct, HPRD, and BIND (Supplementary Table S5A,B). Analysis of these datasets revealed reciprocal binary interactions, with FGFR3_S444 predicted to interact with FGFR3_S445, and vice versa, suggesting potential intramolecular regulatory crosstalk between these adjacent sites. In addition, STX4_S117 was identified as a positively co-modulated complex interactor of FGFR3_S445, based on evidence from IntAct. For FGFR4_S573, we identified 16 positively co-modulated binary interactors and one negatively co-modulated binary interactor (CCDC85C_S258) (Table 2). Furthermore, four proteins were identified as positively co-modulated complex interactors of FGFR4_S573. There were no significant data on negatively co-modulated complex interactors of FGR3_S444, S445, and FGFR4_S573 (Table 2).

### 3.7. Potential Upstream Kinases and Phosphatases of FGFR3and FGFR4 Predominant Sites

**Figure 6:**
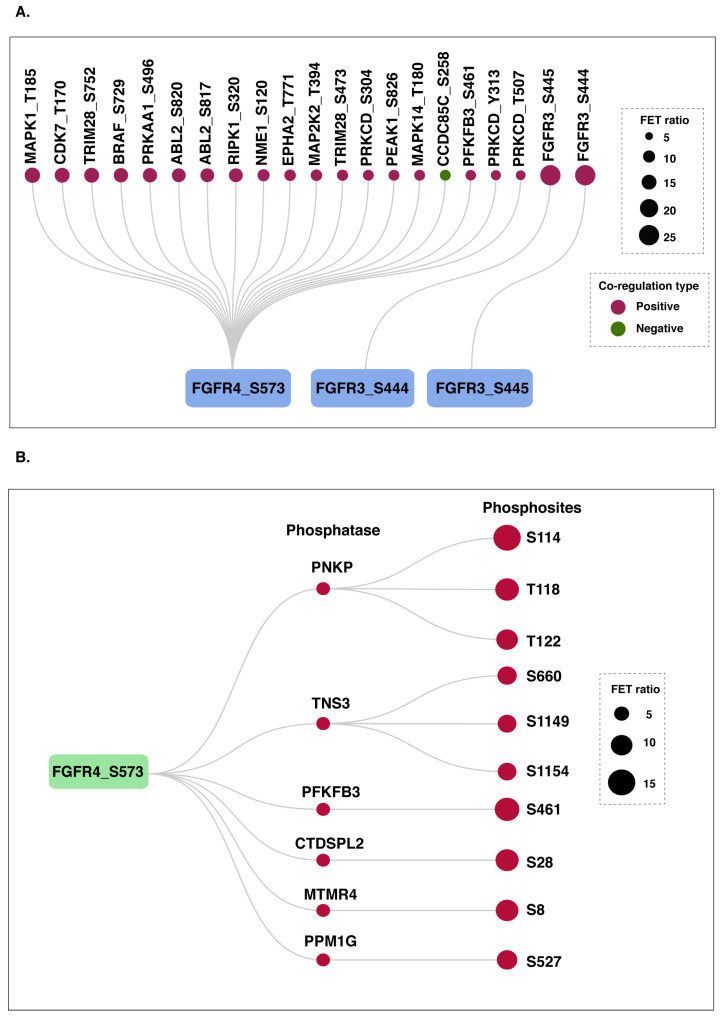
Co-modulation-based identification of potential upstream kinases and phosphatases regulating FGFR3 and FGFR4 phosphorylation. (**A**) The kinases that showed positive and negative co-modulation with FGFR3 and FGFR4 predominant sites are highlighted with different colors. (**B**) Positively co-modulated phosphatases of FGFR4_S573. The size of the nodes represents the FET ratio.

The binding of FGFRs with their ligands induces their dimerization and activates intrinsic kinase activity through the autophosphorylation of tyrosine residues within the intracellular kinase domains [1]. To date, no upstream kinases apart from FGFR family members have been reported. Using co-modulation analysis, we identified positive co-modulation of ten kinases with the FGFR4 at S573, whereas one kinase exhibited negative co-modulation. Among the positive co-modulated kinases, MAPK1 was identified as the possible upstream kinase of FGFR4 by the kinase prediction tool NetworKIN. In addition, the co-modulation analysis highlighted a reciprocal phosphorylation relationship between FGFR3_S444 and FGFR3_S445, in which each was identified as a positively co-regulated kinase for the other (Figure 6A). Furthermore, potential phosphatases were identified exclusively for FGFR4_S573, all of which showed evidence of positive co-modulation with predominant phosphosite. These candidates include PNKP, TNS3, PFKFB3, and CTDSPL2, among others (Figure 6B).

### 3.8. Differential Abundance of FGFR3 and FGFR4 Phosphosites Across Cancer Types

**Figure 7:**
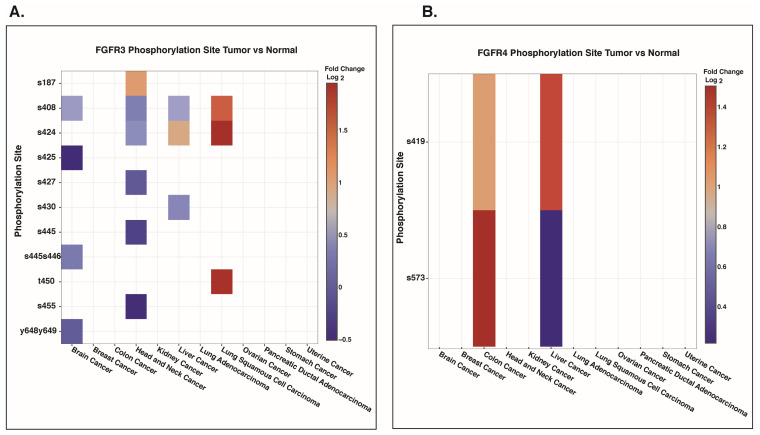
Data from Cprosite shows the differential abundance of FGFR3 and FGFR4 phosphosites in various cancers. Phosphosite level abundance of (**A**) FGFR3 and (**B**) FGFR4 in multiple cancers.

To investigate the cancer-specific expression patterns of FGFR3 and FGFR4 phosphosites, we analyzed data from the CProSite database. The predominant FGFR3 phosphosites, S444 and S445, were detected exclusively in head and neck cancers, where both sites exhibited reduced phosphorylation levels (S444: log_2_FC = −0.19; S445: log_2_FC = −0.45). In contrast, other FGFR3 phosphosites, including T450 (log_2_FC = 1.92), S424 (log_2_FC = 1.95), and S408 (log_2_FC = 1.50), showed markedly increased abundance in lung squamous cell carcinoma (Figure 7A). Notably, the predominant FGFR4 phosphosite S573 displayed elevated phosphorylation in colon cancer (log_2_FC = 1.50) and a modest increase in liver cancer (log_2_FC = 0.22). In addition, FGFR4 S419 also demonstrated increased phosphorylation in both colon cancer (log_2_FC = 1.03) and liver cancer (log_2_FC = 1.33) (Figure 7B).

### 3.9. Disease Associations of Proteins Co-Modulated with FGFR3 and FGFR4

**Figure 8:**
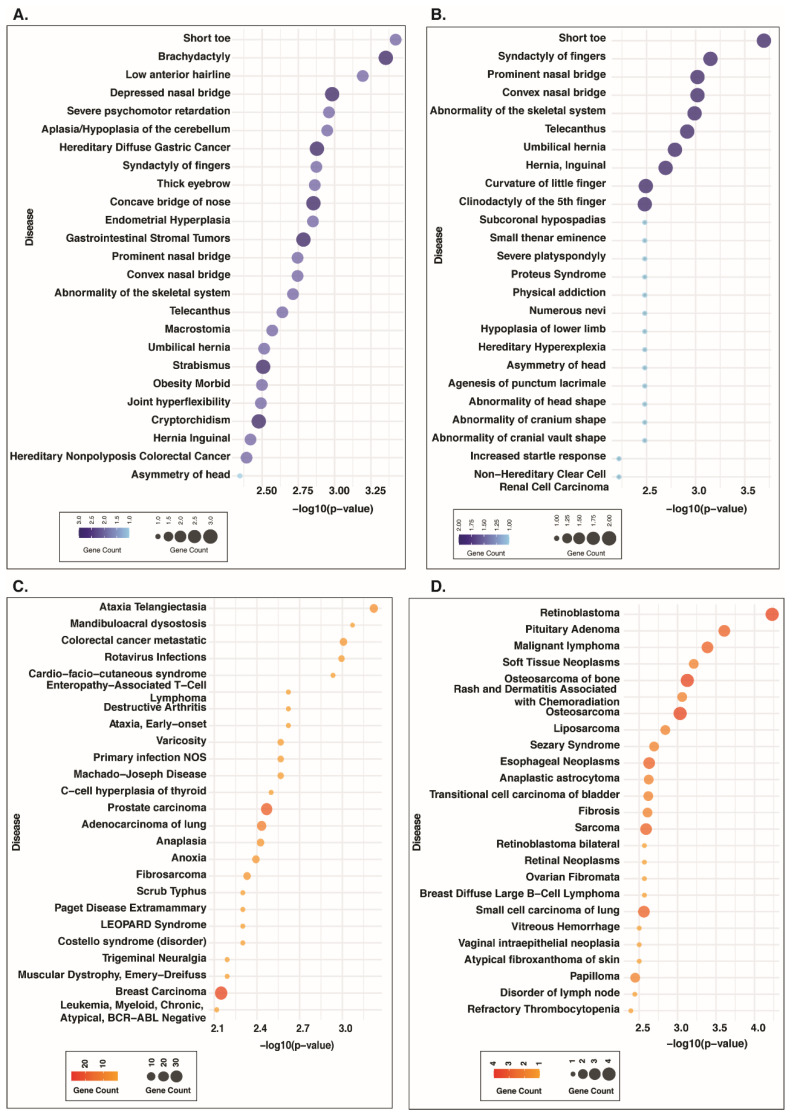
FGFR3 and FGFR4 co-modulated phosphoproteins are predominantly associated with fetal developmental disorders. The bubble plot depicts the diseases enriched for proteins positively co-modulated with FGFR3 (**A**) S444 and (**B**) S445. Diseases enriched for proteins (**C**) positively and (**D**) negatively co-modulated with FGFR4_S573.

The disease enrichment analysis of proteins co-modulated with FGFR3 and FGFR4 was performed using DisGeNET. Proteins positively co-modulated with FGFR3_S444 were found to be associated with brachydactyly (SPECC1L; ARID1A; FGFR3), short toe (SPECC1L; FGFR3), low anterior hairline (ARID1A; FGFR3), hereditary diffuse gastric cancer (CTNNA1; SYMPK; ARID1A), and gastrointestinal stromal tumors (CTNNA1; ARID1A; FGFR3) (Figure 8A, Supplementary Table S6A). The proteins co-modulated with FGFR3_S445 were enriched in short toe (SPECC1L; FGFR3), syndactyly of fingers (SPECC1L; FGFR3), abnormality of the skeletal system (SPECC1L; FGFR3), and non-hereditary clear cell renal cell carcinoma (PBRM1) (Figure 8B, Supplementary Table S6B). For FGFR4_S573, positively co-modulated proteins were associated with ataxia telangiectasia (NCOR2; TRIM28; PNKP; CCDC6; MAPK1; TOP1; TP53BP1), mandibuloacral dysostosis (SUN2; CCDC6), rotavirus infections (JUN; NUP155; MAPK1), cardio-facio-cutaneous syndrome (ZHX2; MAPK1), prostate carcinoma (ZHX2; JUN; CRYBG1; NCOA3; PRKCD; CAD; ATAD2; SGTA; ADAR; FOXK1; NCOR2; PDLIM2; CTTN; FASN; GIPC1; MAPK1; SPRY1; TOP1; TP53BP1; TNS3; NELFE), and breast carcinoma (SLC20A1; HTT; ADAR; PPP1R9B; SART1; TRIM28; PIEZO1; MAPK1; TP53BP1; TNS3; ARFGEF3; NELFE; RBM39; ZHX2; JUN; NCOA3; PRKCD; CAD; ATAD2; MYO9B; NCOR2; CTTN; FASN; GIPC1; CCDC6; TAGLN2; SPRY1; TOP1) (Figure 8C, Supplementary Table S7A). Negatively co-modulated proteins of FGFR4_S573 were involved in retinoblastoma (RB1; WEE1; SATB1; MKI67), pituitary adenoma (RB1; WEE1; MKI67), malignant lymphoma (RB1; WEE1; MKI67), and osteosarcoma of bone (RB1; WEE1; SATB1; MKI67) (Figure 8D, Supplementary Table S7B).

## 4. Discussion

FGFRs are membrane-bound receptor tyrosine kinases that play a pivotal role in regulating cell proliferation, differentiation, migration, and survival. FGFR signaling is critical for the normal development of organs in the digestive, nervous, and circulatory systems, and also plays a key role in maintaining tissue homeostasis [7]. Our organ-level expression analysis of FGFR1–5 revealed tissue-specific RNA expression of FGFR2 in the brain, FGFR3 in the skin, and FGFR4 in the liver. In contrast, FGFR1 and FGFR5 exhibited broad expression across nearly all human organs. The enrichment of FGFR2 transcripts in the brain, aligns with its established involvement in neurodevelopment, while aberrant FGFR2 signaling has been implicated in craniofacial and neurodevelopmental disorders [61]. The skin-specific expression of FGFR3 is consistent with its role in epidermal differentiation and provides a molecular basis for the strong association between FGFR3 mutation in seborrheic keratoses and epidermal nevi [62,63].

Elevated gene and protein expression, along with the increased phosphorylation of FGFR1-4, has been associated with multiple malignancies, including breast cancer, hepatocellular carcinoma, ovarian cancer, and lung adenocarcinoma [59,64,65]. Dysregulation of FGFR signaling disrupts normal tissue and organ development and contributes to skeletal disorders, including achondroplasia and thanatophoric dysplasia (primarily linked to FGFR3 mutations), craniosynostosis syndromes (associated with FGFR2 mutations), as well as muscle disorders such as muscular dystrophy [7,66,67]. Owing to their critical role in cancer and developmental disorders, we aimed to explore the phosphorylation landscape of the FGFR family of proteins by integrating the data from multiple phosphoproteomics studies.

Phosphorylation of FGFRs is essential for the activation of their kinase activity and for stimulating downstream signaling through the phosphorylation of effector proteins. Phosphoproteomics, therefore, represents a powerful approach for characterizing protein phosphorylation signatures across diverse biological and disease contexts. By integrating and systematically analyzing large-scale public phosphoproteomics datasets, the present study identified several phosphosites within FGFRs that show predominant differential abundance. These include Y653 in FGFR1; S453, Y586, Y656, and Y657 in FGFR2; S444 and S445 in FGFR3; and S573 in FGFR4. These predominant sites are mainly located within the kinase domains of FGFR1-4, except for S453 in FGFR2 and S444/445 in FGFR3, which are located outside the kinase domain. Y653 is an autophosphorylation site in FGFR1, and its phosphorylation enhances FGFR1 kinase activity, leading to activation of the AKT signaling pathway and thereby promoting chemotaxis and migration in endothelial cells [68,69]. Overexpression and increased phosphorylation of FGFR2 at Y653/654 have been reported in gastric cancer. Inhibiting phosphorylation at Y653/654 reduces FGFR2 kinase activity, leading to decreased cell proliferation and increased apoptosis in gastric cancer cells [70,71]. The disease relevance of the predominant sites in FGFR3 (S444 and S445) and FGFR4 (S573) remains unexplored, highlighting the need for further studies to investigate their functional roles.

To further understand the functional diversity of FGFRs, we analyzed the distribution of phosphosites across their isoforms. Although FGFRs are known to exist in multiple isoforms, the datasets used in this study provided phosphosite information for only a limited number of isoforms. For FGFR1, three isoforms such as isoforms 1, 6, and 21 were identified out of 21 known isoforms. In FGFR2, phosphosites were detected in isoforms 1, 16, and 21, among a total of 17 isoforms. For FGFR3, three isoforms (isoforms 1, 2, and 4) were detected out of four known isoforms. For FGFR4, phosphosite information was detected only for the canonical isoform. This limited isoform coverage reflects the inherent limitations of mass spectrometry-based phosphoproteomics, which identifies proteins based on only a small fraction of peptide sequences and therefore cannot reliably distinguish between closely related isoforms [72]. This limitation is particularly important because FGFR isoforms have distinct biological roles, as they differ in ligand binding, and downstream signaling. Isoform switching of FGFR2 has been reported in breast and gastric cancers. For instance, the FGFR2 splice variant C3 was found to be upregulated in cancer cells, and the absence of the 770YXXL motif in C3 leads to reduced receptor internalization, increased receptor activation, and enhanced FRS2-mediated signaling, thereby promoting cancer progression [73]. Another study reported that FGFR2 IIIb C2 is expressed in breast cancer cells, whereas the FGFR2 IIIc isoform is found in invasive breast cancer, with IIIc expression associated with loss of epithelial markers and gain of mesenchymal markers [74]. Additionally, increased expression of the FGFR1 IIIc isoform has been linked to high-grade astrocytomas, ovarian cancer, oral squamous cell carcinoma, bladder cancer, and non-small cell lung cancer [75]. This underscores the need to integrate advanced data-analysis pipelines with conventional bottom-up proteomic strategies to enable reliable phosphosite identification at the proteoform level. Nevertheless, our analysis revealed several phosphosites that are unique to specific FGFR isoforms, as well as sites that are conserved across isoforms. Further investigation is required to clarify the mechanisms underlying isoform-specific modulation and the functional implications of this conservation across FGFRs.

Since the biological functions of phosphosites in FGFRs are not yet fully characterized, we applied a co-modulation analysis to investigate the roles of predominant phosphosites. Co-modulated proteins often show similar functions under specific biological conditions, suggesting involvement in shared signaling pathways or pathway cross talks [76]. Studying these co-modulation patterns can provide insights into phosphosite-specific functions of FGFRs. Hence, we examined the phosphorylation patterns on other proteins that are co-modulated with the predominant sites on FGFR3 and FGFR4 (significantly co-modulated were not found for FGFR1 and 2). Among the top positively co-modulated PsOPs of FGFR3, GPHN (S194) and MYH10 (S1956) were the most highly co-modulated. GPHN is a scaffolding protein primarily found at the postsynaptic membranes of inhibitory synapses, playing a central role in clustering GABAA and glycine receptors. It is also involved in the recruitment of neuroligin-2 to inhibitory synapses. Dysregulation of GPHN was reported in autism spectrum disorder, schizophrenia, and epilepsy [77]. MYH10 encodes a non-muscle myosin heavy chain II isoform that functions as a motor protein. It is primarily expressed in non-muscle cells, where it regulates cell morphology, cell adhesion, and migration [78]. The S1956 phosphorylation on MYH10 was associated with cytoskeletal reorganization [79]. MYH10 is essential for alveologenesis, and its reduced abundance impairs extracellular matrix remodeling, as well as cell proliferation and differentiation, ultimately contributing to respiratory distress [80]. PNKP_T118/122 and H1-4_S2 were the highly positively co-modulated sites with FGFR4_S573. PNKP acts as a DNA repair enzyme with dual functions, serving as both a kinase and a phosphatase. The T118 and T122 residues of PNKP were found to be hyperphosphorylated following UV-induced DNA damage [81]. H1-4 is a member of the linker histone H1 family, which is essential for stabilizing higher-order chromatin structure. H1-4 in phosphorylated form functions as a transcriptional activator [82]. For FGFR4_S573, the top negatively co-modulated sites include PRP4K_S277 and RB1_S811. PRP4K is a haploinsufficient tumor suppressor gene, and its reduced abundance has been linked to aggressive breast and ovarian cancers, characterized by enhanced migratory and invasive properties [83]. RB1 is a studied tumor suppressor gene and plays a pivotal role in the regulation of the cell cycle. The phosphorylation of RB1 at S811 was associated with carcinogenesis and cell growth [49]. Altered expression and activation of RB1 were reported in various cancers, including breast cancer, prostate cancer, colon adenocarcinoma, and thyroid cancer [84]. However, the phosphosite-specific functions of GPHN_S194, MYH10_S1956, H1-4_S2, and PRP4K_S277 remain uncharacterized and warrant further investigation.

It is crucial to identify the upstream kinases responsible for the phosphorylation of FGFR3/4, as they are involved in multiple diseases. Currently, no kinases outside the FGFR family are known to phosphorylate them. Our kinase analysis identified several co-modulated kinases for FGFR4, with MAPK1 being highlighted as a candidate kinase for FGFR4_S573, supported by additional evidence from the kinase prediction tool NetworKIN. The phosphorylation of MAPK1 at T185 induces its enzymatic activity [85]. Moreover, MAPK1 has been identified as a putative kinase for FGFR1 and FGFR2 [86,87]. These observations collectively point to MAPK1 as a potential upstream kinase for FGFR4. However, this warrants further experimental validation. For FGFR3_S444, the kinase predicted was FGFR3_S445, and reciprocally, this observation aligns with the established mechanism of intramolecular phosphorylation within FGFRs. Additionally, we also identified potential phosphatases of FGFR4_S5734, such as PNKP, TNS3, PFKFB3, and CTDSPL2, that were known to be involved in carcinogenesis, cell motility, cell growth, DNA repair, and apoptosis [88,89].

To understand the disease association of predominant sites of FGFR3 and FGFR4, we performed disease enrichment analysis of phosphoproteins co-modulated with these proteins. The analysis revealed the proteins co-modulated with FGFR3 were mainly involved in diseases such as brachydactyly, short toe, syndactyly of fingers, and abnormality of the skeletal system, which align with the well-established role of FGFR3 in skeletal dysplasias [90,91]. The enriched disease associated with phosphoproteins co-modulated with FGFR4 included several cancers, such as colorectal cancer metastasis, retinoblastoma, pituitary adenoma, malignant lymphoma, and osteosarcoma of bone. These results are in line with earlier studies that have demonstrated the involvement of FGFR4 in cancer development and progression [92].

In the present study, we emphasized the phospho-modulation of FGFR3 and FGFR4 along with their co-modulated proteins, underscoring their associations with various diseases. These findings provide important insights into phosphorylation-dependent regulatory mechanisms of FGFRs, offering a framework to better understand their contribution not only to oncogenic signaling but also to broader cellular processes. Such information can serve as a foundation for future investigations aimed at delineating their functional roles and potential as therapeutic targets.

## 5. Conclusions

The present study provides a comprehensive overview of the phosphorylation network of the FGFR family proteins, with particular emphasis on FGFR3 and FGFR4. Through systematic analysis of human cellular phosphoproteomics datasets, we identified predominant phosphosites across the family, including Y653, Y583, and Y654 in FGFR1; Y656 in FGFR2; S444 and S445 in FGFR3; and S573 in FGFR4. In addition, we observed differential abundance of key predominant sites, specifically Y653 in FGFR1; S453, Y586, Y656, and Y657 in FGFR2; S444 and S445 in FGFR3; and S573 in FGFR4. By utilizing co-modulation analysis strategy, the phosphosite specific biological functions of FGFR3 and FGFR4 were elucidated. This study represents the first investigation into the co-modulated proteins of FGFR3 and FGFR4 at the phospho level, identifying 32 and 89 significantly co-modulated PsOPs for FGFR3 and FGFR4, respectively. Phosphosites co-modulated with FGFR3 were associated with functions related to cytoskeletal organization, cell motility, and carcinogenesis. Likewise, phosphosites co-modulated with FGFR4 were linked to cytoskeletal organization, cell motility, and carcinogenesis. Beyond the upstream kinases from the FGFR family, we also identified MAPK1 as a potential upstream kinases for FGFR4. Further, disease enrichment analysis revealed that proteins co-modulated with FGFR3 were primarily involved in skeletal developmental disorders, whereas those associated with FGFR4 were linked to various cancers. In summary, our findings provide insights into the phosphosite-specific roles of FGFR and their co-modulated proteins across different biological processes. Nonetheless, further experimental studies are required to confirm the functional relevance of PsOPs co-modulated with the FGFR predominant site and to explore their potential as a therapeutic target.

## 6. Limitations and Future Outlook

The present study carries certain inherent limitations, as it relied on multiple phosphoproteomics datasets to investigate the phosphosite co-modulation of FGFRs. The datasets utilized in this study possess differences in sample preparation, experimental methods, analytical platforms, and biological contexts. While our approach offers an extensive overview of FGFR phosphorylation in the canonical isoform, the phosphorylation information for other isoforms remains limited due to the inherent difficulty of conventional bottom-up proteomics in accurately capturing proteoform-specific modification patterns.

Despite these limitations, our in silico approach offers testable hypotheses for experimentally validating the role of FGFRs phosphorylation events in various diseases. Experimental strategies, such as site-directed mutagenesis, could be used to determine the functional contribution of specific predominant sites to receptor activation, downstream signaling, and disease phenotypes. Phospho-specific antibodies can be used to evaluate the differential abundance of FGFRs predominant phosphosites in cell lines, animal models, and patient-derived samples of skeletal disorders and cancers. Additionally, the predicted upstream kinases of FGFR4, such as MAPK1, can be validated using the in vitro kinase and phosphatase assays and kinase/phosphatase inhibitors. CRISPR/Cas9-mediated phosphosite editing could be used to assess the functional role of predominant phosphosites identified in FGFRs in development of skeletal disorders and cancers. Furthermore, integrating these approaches into patient-derived samples, organoids, and disease-specific animal models would enable direct correlation of the predicted phosphoregulatory networks with clinically observed phenotypes, thereby establishing mechanistic links between the computational findings and human disease.

## Figures and Tables

**Table 1:** Phosphosites conserved across the fibroblast growth factor receptor 1-3 (FGFR1-3) isoforms.

Aminoacid Sequence	Phosphosite Position in Isoforms		
	FGFR1 Canonical Isoform	FGFR1 Isoform-6	FGFR1 Isoform-21
LVRPSRLS(p)SSGTPML	S450	_	_
PSRLSS(p)SGTPMLAGVSEYELPEDPR	S451	_	_
PSRLSSS(p)GTPMLAGVSEYELPEDPR	S452	_	_
RPPGLEYCYNPS(p)HNPEEQLSSK	S588	_	_
RPPGLEY(p)CYNPSHNPEEQLSSK	Y583	_	Y614
RPPGLEYCY(p)NPSHNPEEQLSSK	Y585	_	Y616
DLVSCAY(p)QVARGMEYLASK	Y605	_	Y636
DLVSCAYQVARGMEY(p)LASK	Y613	_	_
DIHHIDY(p)YKK	Y653	Y491	Y684
DIHHIDYY(p)K	Y654	_	Y685
GNLREY(p)LQAR	_	_	Y603
MDKPSNCTNELY(p)MMMR	_	_	Y761
PSRLSSSGTPMLAGVSEY(p)ELPEDPR	_	_	Y494
	**FGFR2 Canonical** **Isoform**	**FGFR2** **Isoform-16**	**FGFR2** **Isoform-21**
VRITTRLS(p)STADTPM	S452	_	_
RITTRLSS(p)TADTPML	S453	S454	_
KDLSDLVS(p)EMEMMKM	S533	-	_
RPPGMEYS(p)YDINRVP	S587	S588	_
SQPLEQYS(p)PSYPDTR	S780	_	_
NTPLVRIT(p)TRLSSTA	T448	_	_
ITTRLSST(p)ADTPMLA	T454	_	_
IGGY(p)KVR	Y207 *	_	_
MLAGVSEY(p)ELPEDPK	Y466	_	_
RRPPGMEY(p)SYDINRV	Y586	Y587	_
PPGMEYSY(p)DINRVPE	Y588	_	_
RDINNIDY(p)YKKTTNG	Y656	Y657	_
DINNIDYY(p)KKTTNGR	Y657	Y658	_
LRRQ—VS(p)AESSSSM	_	S432	_
ILT(ph)LTTNEEEK	_	_	T673
ILTLT(ph)TNEEEK	_	_	T675
ILTLTT(p)NEEEK	_	_	T676
	**FGFR3 Canonical** **Isoform**	**FGFR3** **Isoform-2**	**FGFR3** **Isoform-4**
GLGS(p)PTVHK	S408	S410	S408
QVS(p)LESNASMSSNTPLVR	S424	S426	_
QVSLESNASMS(p)SNTPLVR	S427 *	_	S427
QVSLESNASMS(p)SNTPLVR	S432	_	_
IARLS(p)SGEGPTLANVSELELPADPK	S444	S446	S444
IARLSS(p)GEGPTLANVSELELPADPK	S445	S447	_
RPPGLDYS(p)FDTCKPPEEQLTFK	S578	_	_
KKGLGSPT(p)VHKISR	T410	_	T410
IARLSSGEGPT(p)LANVSELELPADPK	T450	_	T450
DVHNLDY(p)YKK	Y647	_	_

* not reported in PhosphositePlus.

**Table 2:** Co-modulated binary and complex protein interactors associated with predominant phosphosites of FGFR3 and FGFR4.

Predominant Sites	PsOPs	Interaction Type
FGFR3_S444	FGFR3_S445	Binary and complex
FGFR3_S445	FGFR3_S444	Binary and complex
	STX4_S117	Complex
FGFR4_S573	SUGT1_T265	Binary
	SUGT1_S11	Binary
	ABL2_S820	Binary
	MAPK1_T185	Binary
	NUP155_S992	Binary
	ABL2_S817	Binary
	EZR_S539	Binary
	EPN2_S173	Binary
	HLA-A_S359	Binary and complex
	SPRY1_S50	Binary
	EPHA2_T771	Binary and complex
	PDLIM2_S206	Binary
	PEAK1_S826	Binary
	MAPK14_T180	Binary
	PLEKHA1_S380	Binary
	TANC1_S132	Binary
	MEPCE_S254	Complex
	HLA-A_S359	Binary and complex
	TEX2_T262	Complex
	EPHA2_T771	Binary and complex
	CCDC85C_S258	Binary

## Data Availability

The datasets supporting the conclusions of this article are included within the article and its Supplementary Materials.

## Supplementary Materials

The following supporting information can be downloaded at https://www.mdpi.com/article/10.3390/proteomes14010008/s1, Table S1A: Phosphosites in FGFR1 compiled from phosphoproteomics profiling studies; Table S1B: Phosphosites in FGFR2 compiled from phosphoproteomics profiling studies; Table S1C: Phosphosites in FGFR3 compiled from phosphoproteomics profiling studies; Table S1D: Phosphosites in FGFR4 compiled from phosphoproteomics profiling studies; Table S2A: Phosphosites in FGFR1 compiled from phosphoproteomics differential abundance studies; Table S2B: Phosphosites in FGFR2 compiled from phosphoproteomics differential abundance studies; Table S2C: Phosphosites in FGFR3 compiled from phosphoproteomics differential abundance studies; Table S2D: Phosphosites in FGFR4 compiled from phosphoproteomics differential abundance studies; Table S2E: Sequence alignment for FGFR1 isoforms 1–21 using Clustal Omega tool; Table S2F: Sequence alignment for FGFR2 isoforms using Clustal Omega tool; Table S2G: Sequence alignment for FGFR3 isoforms using Clustal Omega tool; Table S2H: Sequence alignment for FGFR4 isoforms using Clustal Omega tool; Table S3A: Phosphosites on other proteins (PsOPs) positively co-modulated with the FGFR3 S444 predominant site; Table S3B: PsOPs positively co-modulated with the FGFR3 S445 predominant site; Table S4A: PsOPs positively co-modulated with the FGFR4 S573 predominant site; Table S4B: PsOPs negatively co-modulated with the FGFR4 S573 predominant site; Table S5A: Phosphosites on positively co-modulated binary and complex interactors of FGFR3 S444 and S445 predominant sites; Table S5B: Phosphosites on positively and negatively co-modulated binary and complex interactors of FGFR4 S573; Table S6A: Disease enrichment of proteins positively co-modulated with FGFR3_S444; Table S6B: Disease enrichment of proteins positively co-modulated with FGFR3_S445; Table S7A: Disease enrichment of proteins positively co-modulated with FGFR4_S573; Table S7B: Disease enrichment of proteins negatively co-modulated with FGFR4_S573. Figure S1: FGFR1–5 exhibit distinct organ-specific expression patterns at both the transcript and protein levels. The organ level protein and RNA expression of (A,B) FGFR 1, (C,D) FGFR2, (E,F) FGFR3, (G,H) FGFR4, and (I,J) FGFR4. Protein expression data is shown for each of the 44 tissues based on knowledge-based annotation. Color-coding is based on tissue groups, each consisting of tissues with functional features in common. The consensus dataset consists of normalized expression (nTPM) levels for 55 tissue types, created by combining the HPA and GTEx transcriptomics datasets.

## Abbreviations

RTKs	Receptor tyrosine kinases
FGFRs	Fibroblast growth factor receptors
PsOPs	Phosphosites on other proteins
SADDAN	Severe achondroplasia with developmental delay and acanthosis nigricans
